# Supplementary material for: Investigating DNA words and their distributions across the tree of life
Source: Comput Struct Biotechnol J. 2025 Nov 22;27:5335–47. doi: 10.1016/j.csbj.2025.11.040 (PMC12686733; doi:10.1016/j.csbj.2025.11.040)
Supplement: Supplementary file 1 — Supplementary material [file mmc1.docx]

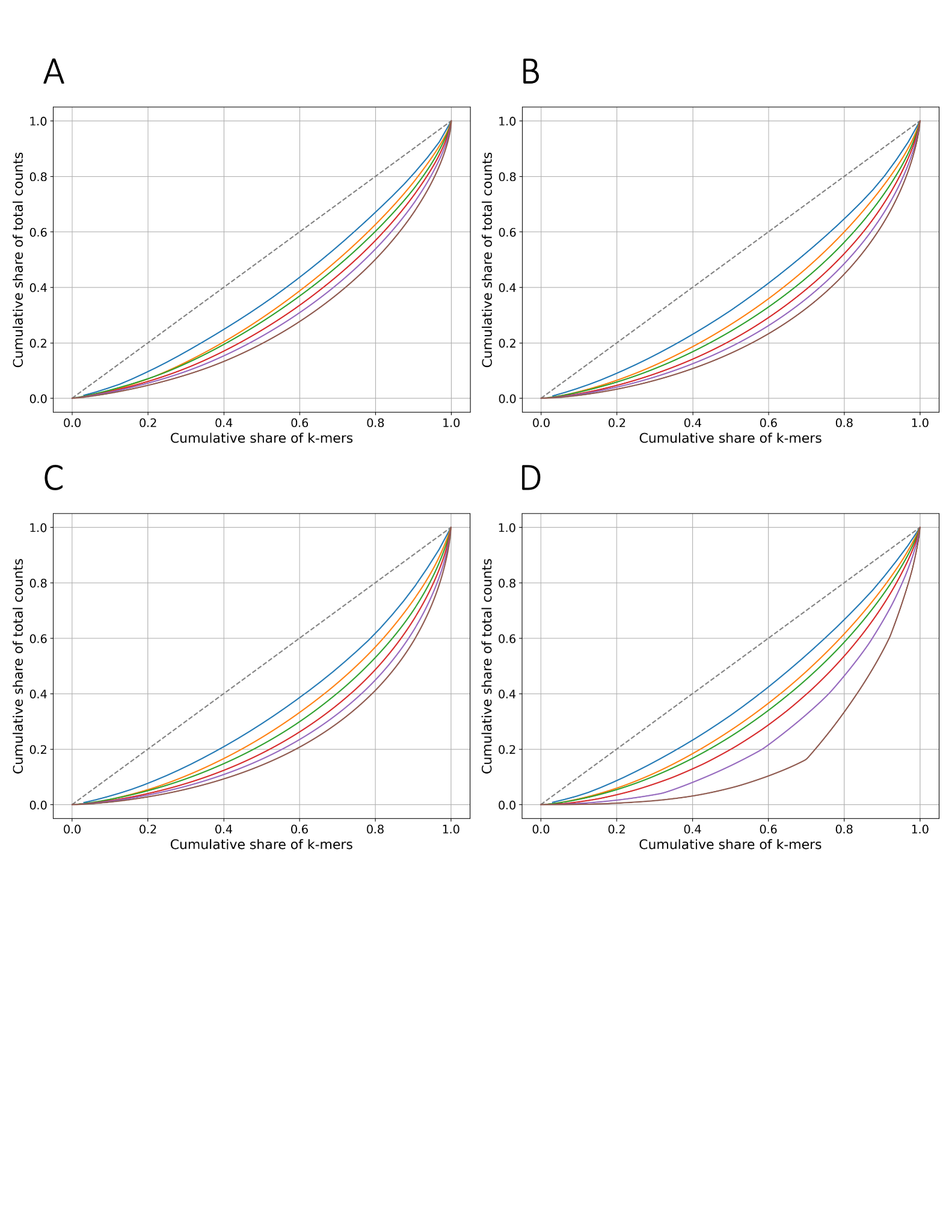


**Supplementary Figure 1.** Lorenz curves illustrate the inequality of k-mer distributions for **A** Eukaryote **B** Bacteria **C** Archaea **D** Viral.


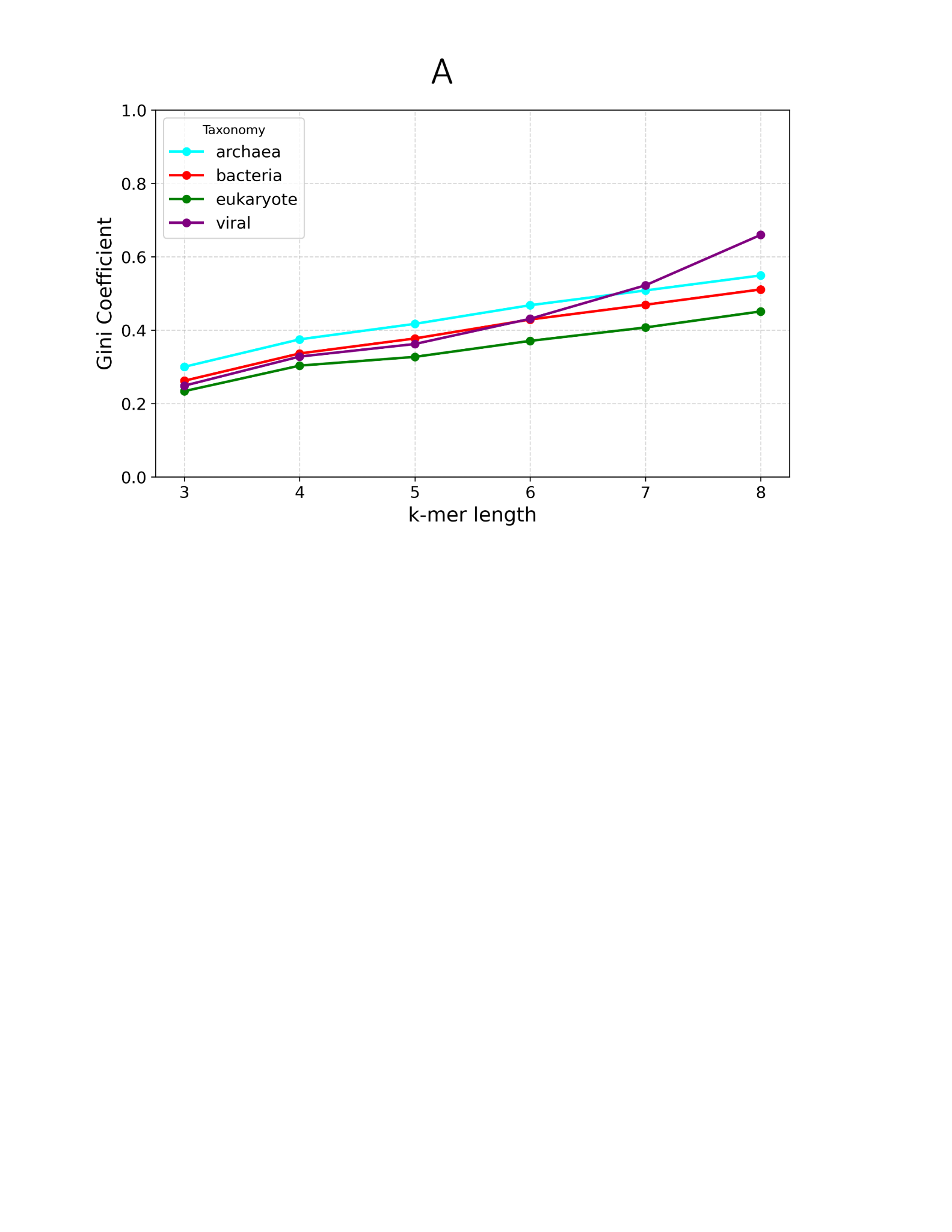


**Supplementary Figure 2:** Gini Coefficient values across taxonomies across k-mer lengths.


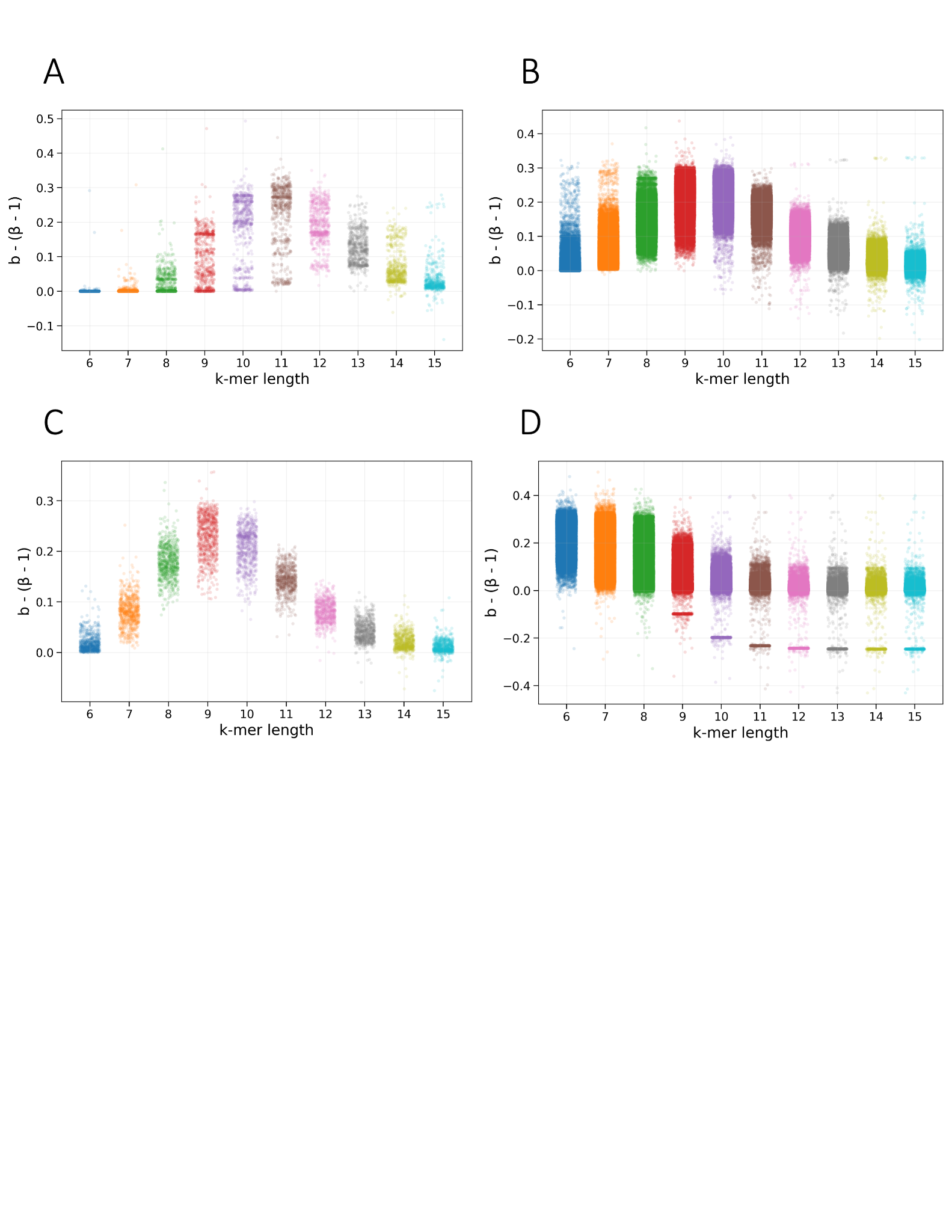


**Supplementary Figure 3: Quantitative comparison of Menzerath and Heaps exponents across taxonomies.** Panels show **(A)** Eukaryotes, **(B)** Bacteria, **(C)** Archaea and **(D)** Viruses. Values of $\Delta=b-(\beta-1)$ clustered near zero indicate agreement between the fitted Menzerath exponent and the Heaps prediction, while systematic positive or negative offsets indicate weaker or stronger Menzerath slopes than expected.

**
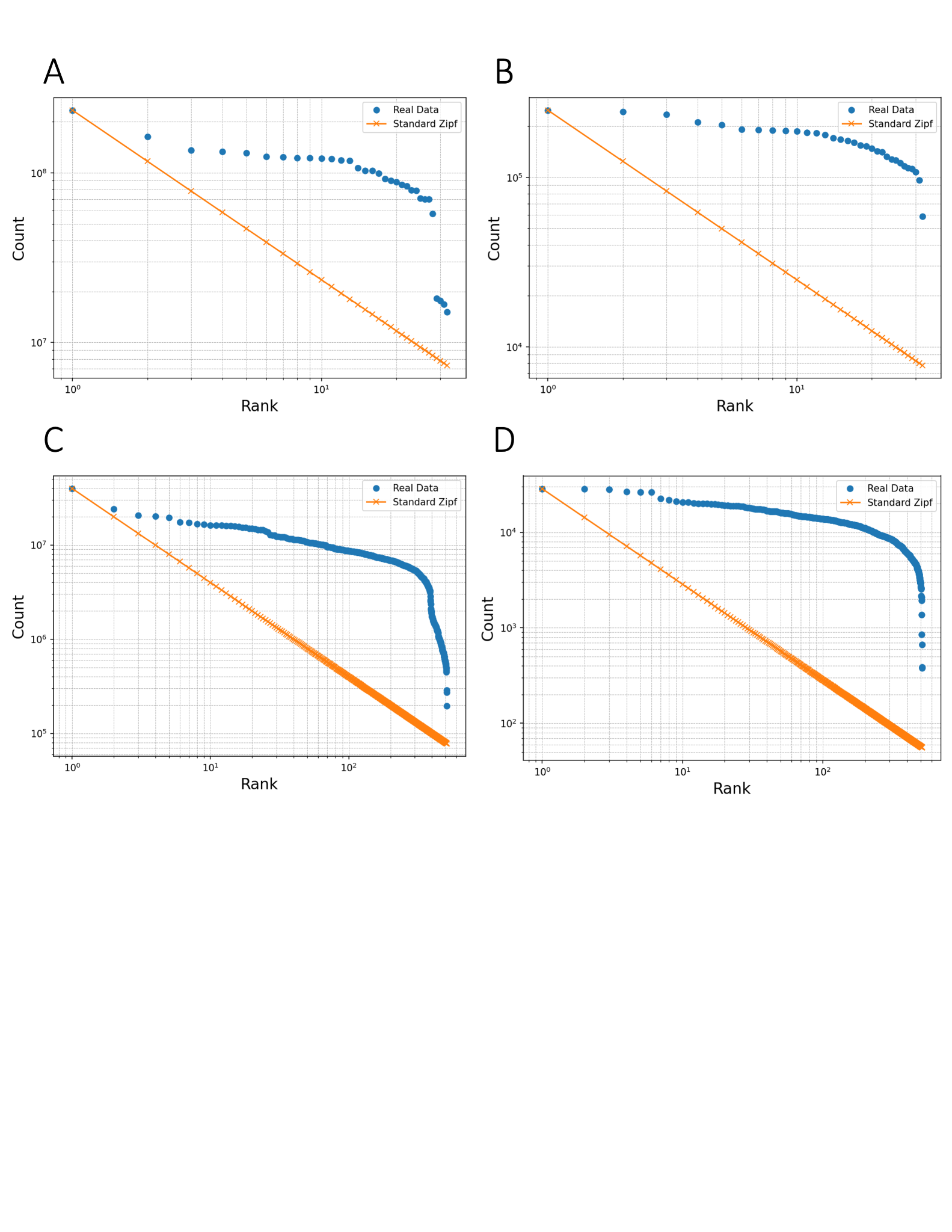
**

**Supplementary Figure 4.** Observed versus theoretical Zipfian distribution for *Homo sapiens* for k=3 and k=5 **A, C** and for *E. coli* for k=3 and k=5 **B, D.**

**
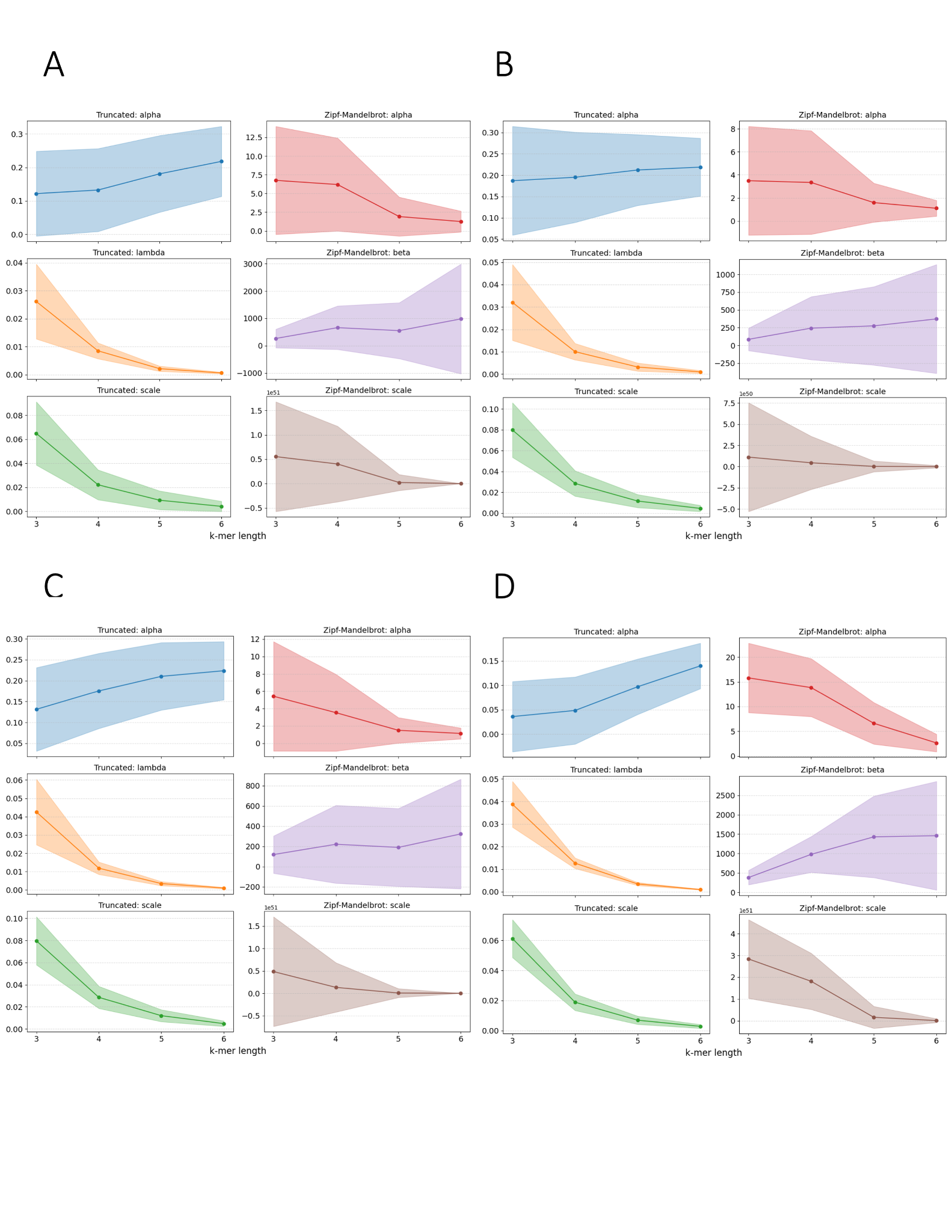
**

**Supplementary Figure 5:** Average fitted parameters for both distributions for k=3,4,5,6**.** The error margins represent 1 std above and below the mean in: **A** eukaryote **B** Bacteria **C** archaea **D** viral.

**
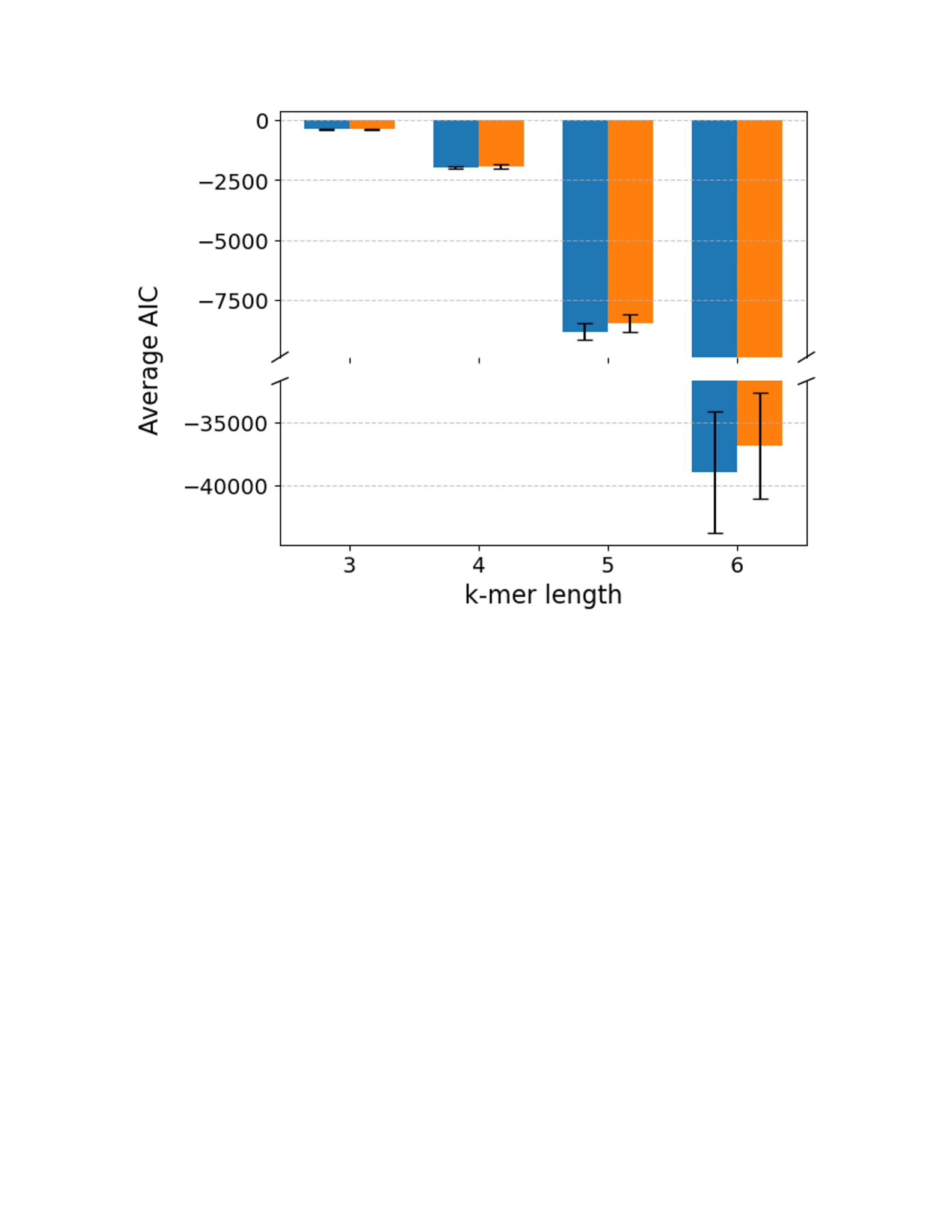
**

**Supplementary figure 6**: **Average Akaike Information Criterion (AIC) for truncated power-law (blue) and Zipf–Mandelbrot (orange) fits to k-mer frequency-rank distributions on a broken y axis.** Error bars denote 1 standard deviation above and below the mean. Lower negative AIC values indicate a better trade-off between goodness-of-fit and model complexity.

**
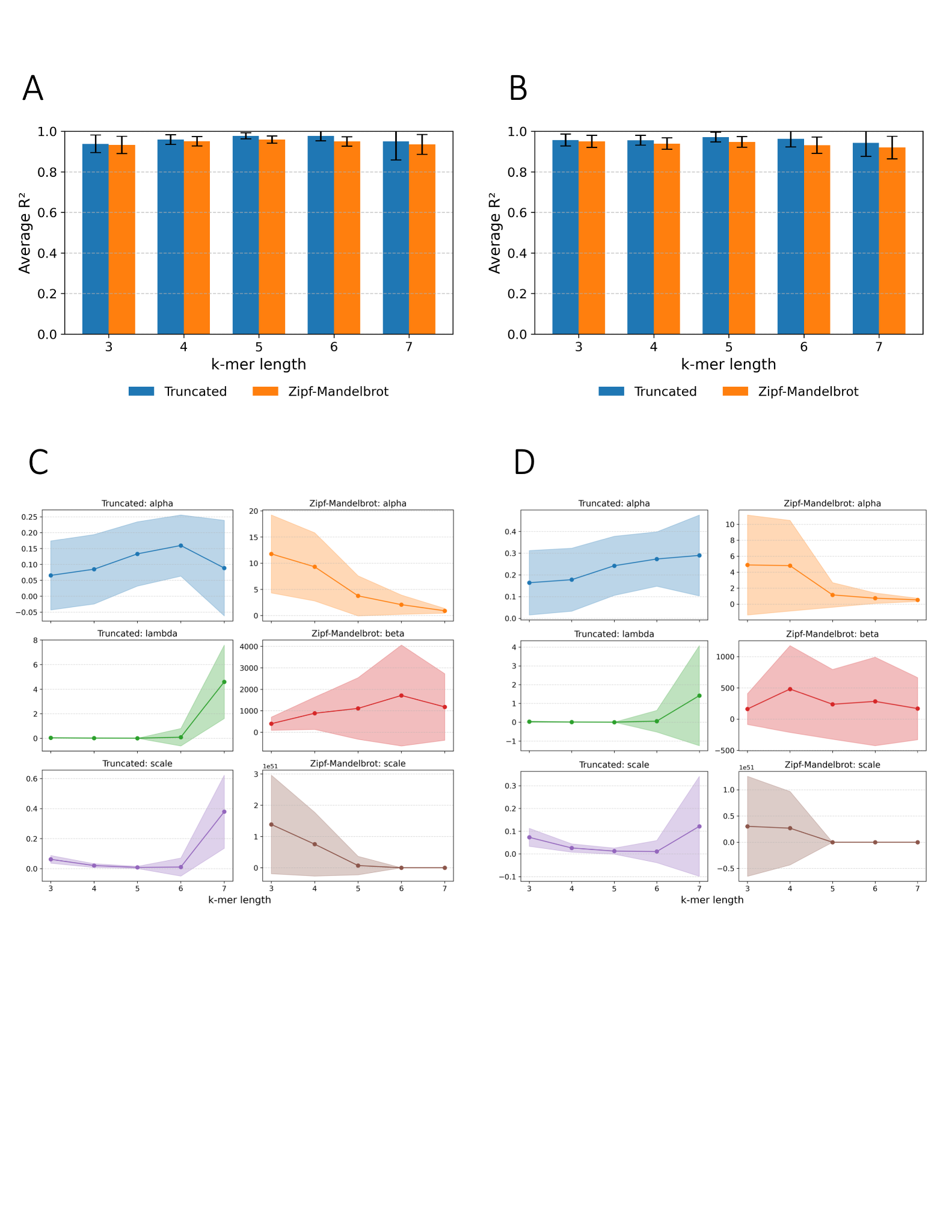
**

**Supplementary Figure 7:** Average $R^{2}$ values for **A coding regions and B non-coding regions** for a subset of the eukaryotic organisms for both distributions across k-mer lengths. Average fitted parameters for both distributions across k-mer lengths. The error margins represent 1 std above and below the mean in: **C coding areas D non-coding areas.**

**
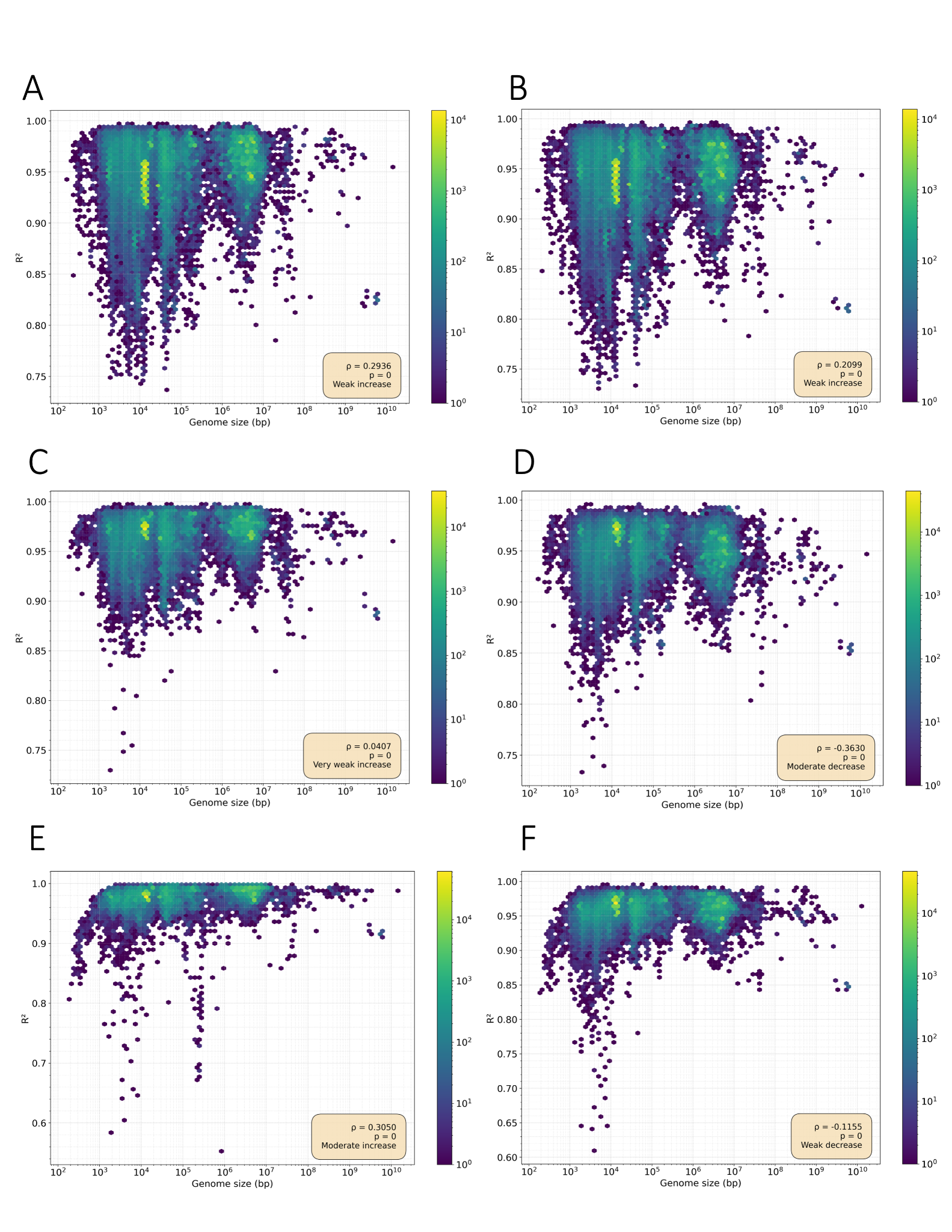
**

**Supplementary figure 8**: R^2^ versus Genome size (log scale) for k=3,4,5 for the truncated power law **(A,C,E)** and the Zipf Mandelbrot **(B,D,F).**

**
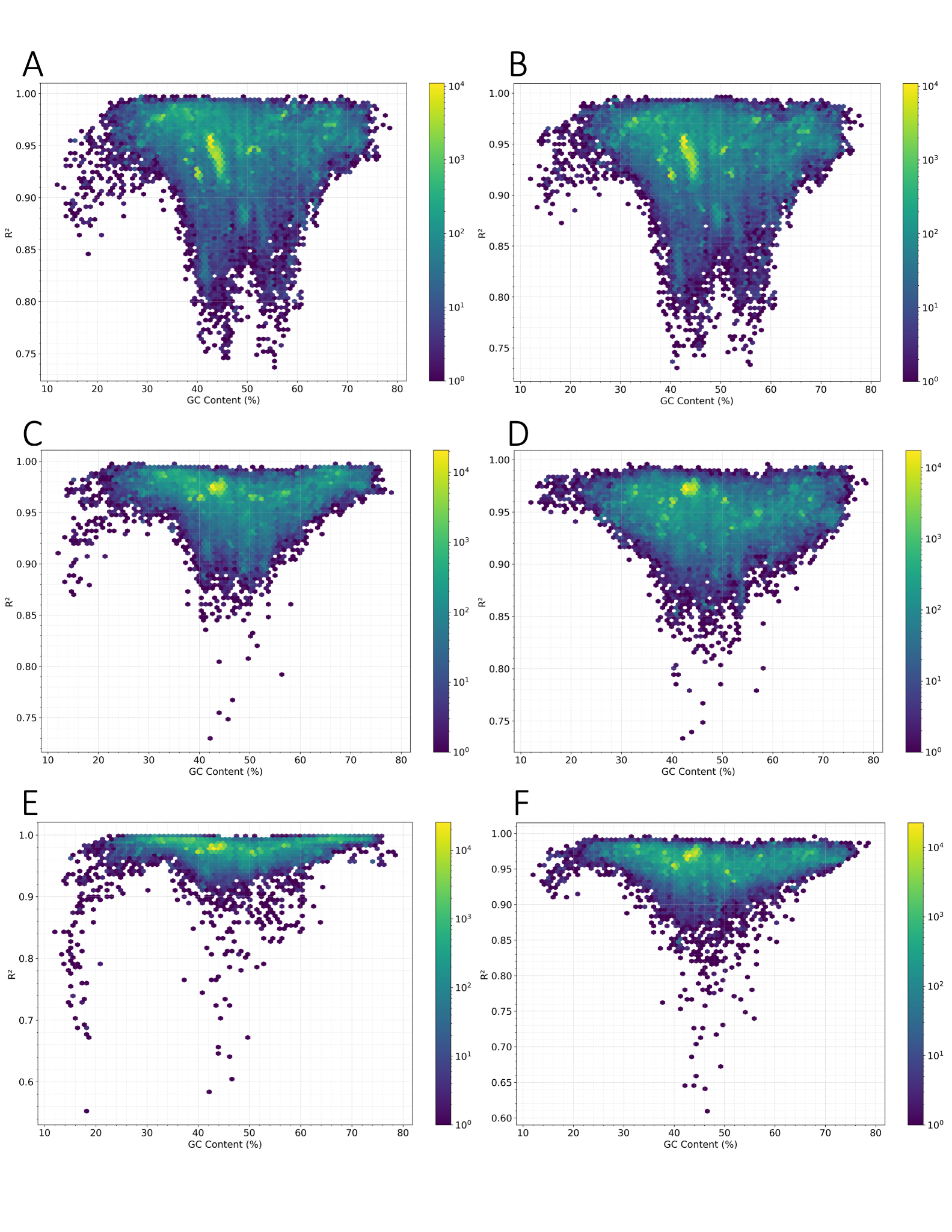
**

**Supplementary figure 9: R^2^ versus GC content for k=3,4,5 for both distributions.** The truncated power law results are shown on the left and the Zipf Mandelbrot results on the right.

**
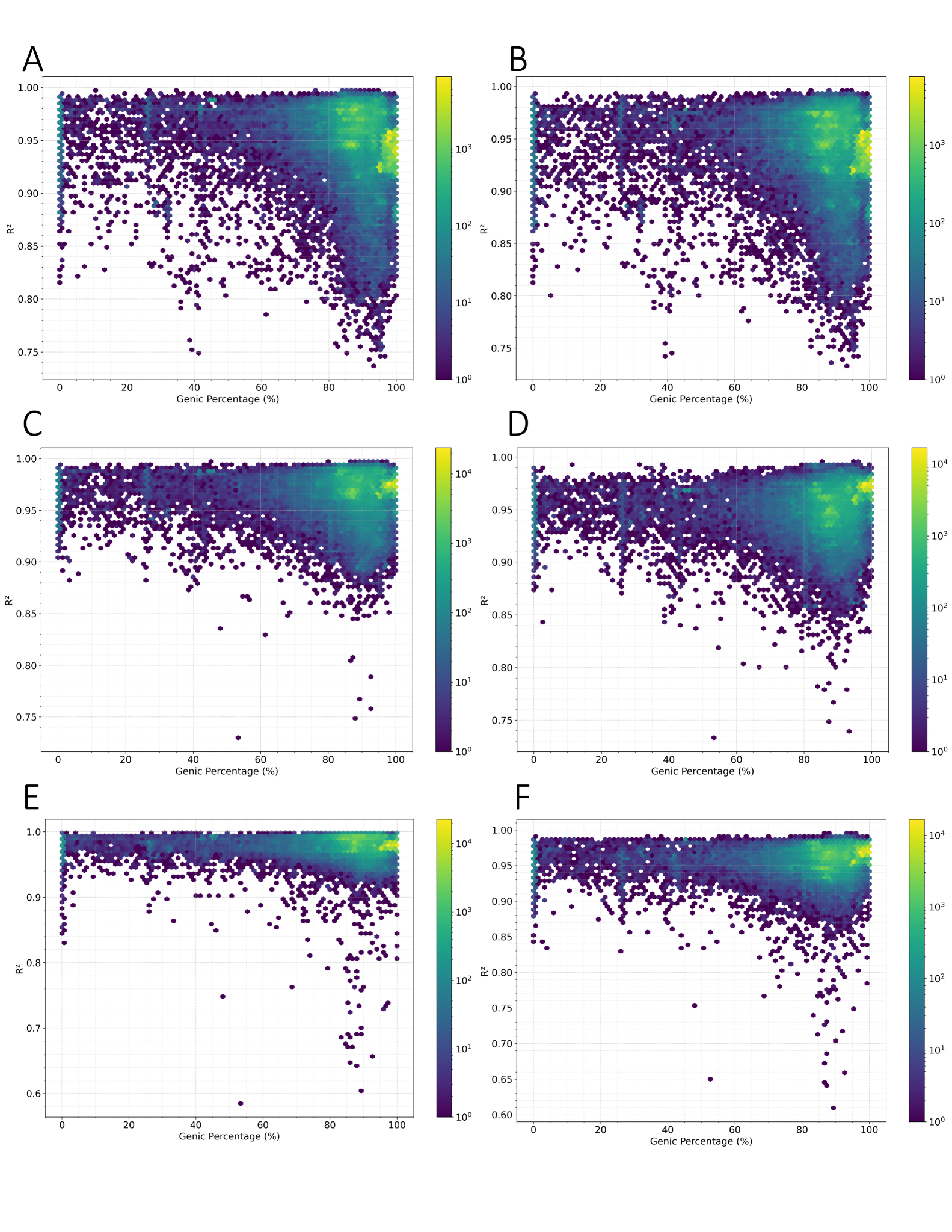
**

**Supplementary figure 10: R^2^ versus Genic percentage for k=3,4,5 for both distributions.** The truncated power law results are shown on the left and the Zipf Mandelbrot results on the right.

**
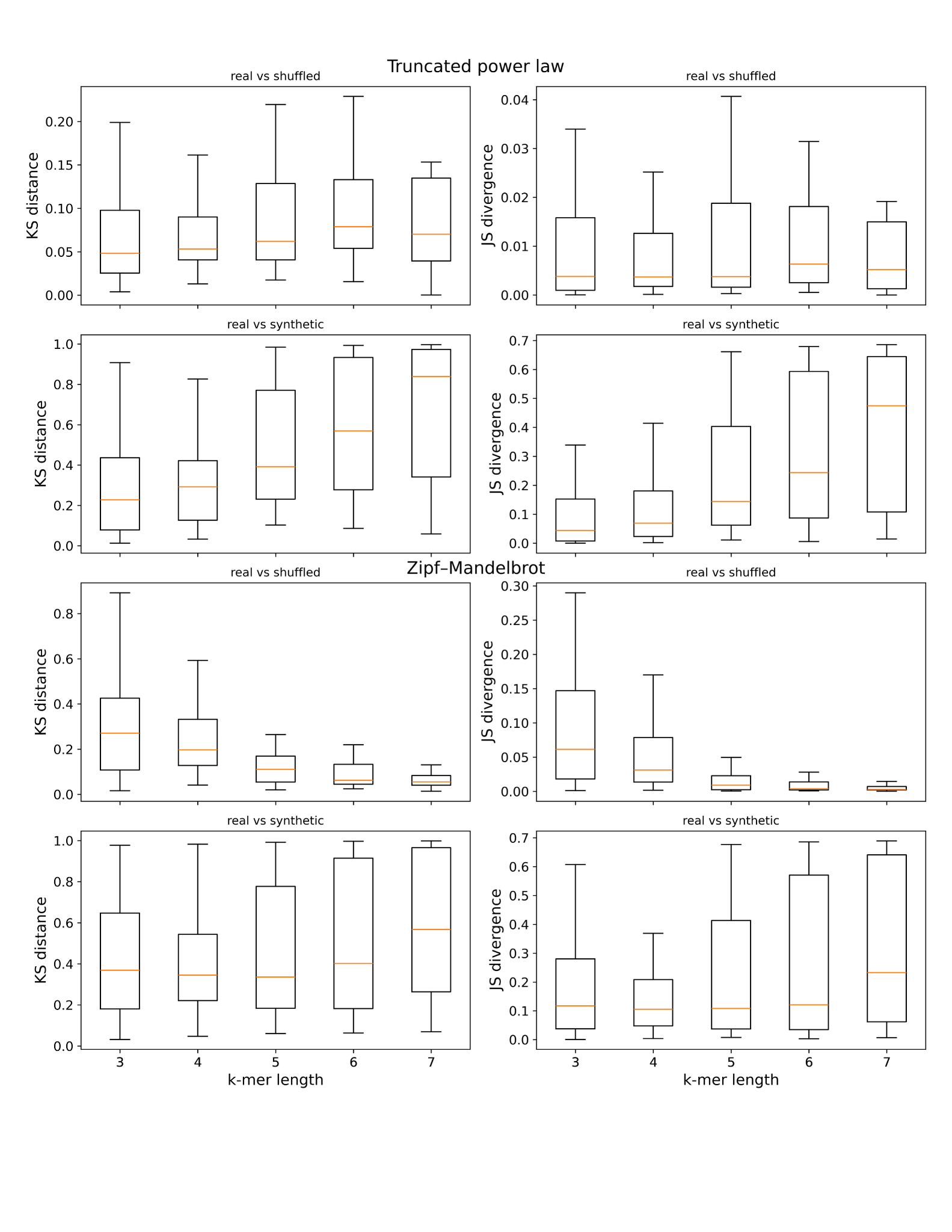
**

**Supplementary Figure 11:** Boxplots show the Kolmogorov-Smirnov (KS) distance (left column) and Jensen-Shannon (JS) divergence (right column) between model predicted rank-frequency curves fitted to real genomes and those fitted to their shuffled and synthetic counterparts, for k-mer lengths k=3-7. The top four panels correspond to the truncated power-law model, and the bottom four panels to the Zipf-Mandelbrot model.

**Supplementary Table 1: Confidence intervals for all three fitted parameters of the Truncated Power law.** We use the normal approximation to demonstrate 95% confidence interval and provide a lower bound, a high bound and their width for each parameter.

| **k** | **α_low** | **α_high** | **α_widt** | **λ_low** | **λ_high** | **λ_widt** | **s_low** | **s_high** | **s_widt** |
| --- | --- | --- | --- | --- | --- | --- | --- | --- | --- |
| **Eukaryotes** | | | | | | | | | |
| **3** | 0.11175 | 0.13188 | 0.02012 | 0.02507 | 0.02720 | 0.00212 | 0.06284 | 0.06703 | 0.00418 |
| **4** | 0.12267 | 0.14235 | 0.01968 | 0.00828 | 0.00873 | 0.00044 | 0.02112 | 0.02310 | 0.00197 |
| **5** | 0.17180 | 0.18998 | 0.01818 | 0.00205 | 0.00219 | 0.00014 | 0.00860 | 0.00983 | 0.00123 |
| **6** | 0.20941 | 0.22664 | 0.01722 | 0.00057 | 0.00062 | 4.72e-05 | 0.00377 | 0.00447 | 0.00069 |
| **Bacteria** | | | | | | | | | |
| **3** | 0.18610 | 0.18817 | 0.00206 | 0.03189 | 0.03217 | 0.00027 | 0.07958 | 0.08001 | 0.00042 |
| **4** | 0.19433 | .19604 | 0.00171 | 0.00998 | 0.01004 | 5.95e-05 | 0.02841 | 0.02861 | 0.00019 |
| **5** | 0.21158 | 0.21293 | 0.00134 | 0.00311 | 0.00314 | 2.93e-05 | 0.01157 | 0.01167 | 0.00010 |
| **6** | 0.21841 | 0.21951 | 0.00109 | 0.00095 | 0.00096 | 1.02e-05 | 0.00453 | 0.00458 | 4.79e-05 |
| **Archaea** | | | | | | | | | |
| **3** | 0.12400 | 0.13880 | 0.0148 | 0.04126 | 0.04392 | 0.00265 | 0.07815 | 0.08138 | 0.00322 |
| **4** | 0.16885 | 0.18217 | 0.01331 | 0.01161 | 0.01211 | 0.00050 | 0.02789 | 0.02938 | 0.00148 |
| **5** | 0.20434 | 0.21630 | 0.01196 | 0.00337 | 0.00354 | 0.00016 | 0.01164 | 0.01244 | 0.00079 |
| **6** | 0.21865 | 0.22897 | 0.01031 | 0.00100 | 0.00106 | 5.49e-05 | 0.00469 | 0.00505 | 0.00036 |
| **Viral** | | | | | | | | | |
| **3** | 0.03544 | 0.03613 | 0.00069 | 0.03871 | 0.03880 | 9.75e-05 | 0.06115 | 0.06127 | 0.00012 |
| **4** | 0.04792 | 0.04858 | 0.00066 | 0.01264 | 0.01266 | 2.23e-05 | 0.01890 | 0.01895 | 5.28e-05 |
| **5** | 0.09673 | 0.09727 | 0.00054 | 0.00346 | 0.00346 | 6.47e-06 | 0.00694 | 0.00696 | 2.62e-05 |
| **6** | 0.13942 | 0.13987 | 0.00044 | 0.00099 | 0.00099 | 1.80e-06 | 0.00286 | 0.00287 | 1.22e-05 |

**Supplementary Table 2: Confidence intervals for all three fitted parameters of the** Zipf-Mandelbrot**.** We use the normal approximation to demonstrate 95% confidence interval and provide a lower bound, a high bound and their width for each parameter.

| **k** | **α_low** | **α_high** | **α_widt** | **β_low** | **β_high** | **β_widt** | **s_low** | **s_high** | **s_widt** |
| --- | --- | --- | --- | --- | --- | --- | --- | --- | --- |
| **Eukaryotes** | | | | | | | | | |
| **3** | 6.1847 | 7.33094 | 1.14624 | 237.79 | 291.772 | 53.9817 | 4.64e+50 | 6.43e+50 | 1.79e+50 |
| **4** | 5.70954 | 6.69655 | 0.98700 | 596.698 | 723.076 | 126.378 | 3.40e+50 | 4.64e+50 | 1.23e+50 |
| **5** | 1.7158 | 2.12859 | 0.41279 | 468.934 | 631.582 | 162.648 | 9.21e+48 | 3.52e+49 | 2.60e+49 |
| **6** | 1.14842 | 1.37046 | 0.22204 | 820.105 | 1139 | 318.897 | -1.2e+41 | 6.03e+41 | 7.28e+41 |
| **Bacteria** | | | | | | | | | |
| **3** | 3.46045 | 3.53678 | 0.07632 | 84.346 | 86.9121 | 2.56613 | 1.06e+50 | 1.17e+50 | 1.03e+49 |
| **4** | 3.30931 | 3.38196 | 0.07264 | 239.854 | 247.033 | 7.17846 | 4.23e+49 | 4.74e+49 | 5.06e+48 |
| **5** | 1.58569 | 1.61296 | 0.02727 | 270.947 | 279.875 | 8.92875 | 2.01e+48 | 3.04e+48 | 1.02e+48 |
| **6** | 1.10332 | 1.11439 | 0.01107 | 367.027 | 379.425 | 12.3979 | 1.10e+47 | 3.37e+47 | 2.27e+47 |
| **Archaea** | | | | | | | | | |
| **3** | 4.95169 | 5.88581 | 0.93411 | 104.777 | 132.144 | 27.3671 | 3.93e+50 | 5.74e+50 | 1.81e+50 |
| **4** | 3.19953 | 3.85576 | 0.65623 | 192.454 | 249.589 | 57.1346 | 9.05e+49 | 1.72e+50 | 8.16e+49 |
| **5** | 1.38662 | 1.60385 | 0.21723 | 161.236 | 218.382 | 57.1467 | -2.0e+48 | 1.26e+49 | 1.46e+49 |
| **6** | 1.08562 | 1.17873 | 0.09310 | 282.863 | 363.245 | 80.3822 | -4.3e+12 | 3.54e+13 | 3.98e+13 |
| **Viral** | | | | | | | | | |
| **3** | 15.7745 | 15.842 | 0.06752 | 380.69 | 382.478 | 1.78836 | 2.83e+51 | 2.84e+51 | 1.73e+49 |
| **4** | 13.8165 | 13.8729 | 0.05639 | 976.392 | 980.839 | 4.44707 | 1.80e+51 | 1.81e+51 | 1.24e+49 |
| **5** | 6.5997 | 6.64011 | 0.04040 | 1425.81 | 1435.94 | 10.121 | 1.55e+50 | 1.60e+50 | 4.84e+48 |
| **6** | 2.64627 | 2.66327 | 0.01699 | 1453.61 | 1467.09 | 13.4778 | 6.61e+48 | 7.46e+48 | 8.43e+47 |

**Supplementary Table 3:** Spearman correlations controlling for genome size and model goodness of fit for both distributions across k-mer lengths. R denotes the Spearman correlation while p are the corresponding p values.

| k | Truncated Power Law | | Zipf-Manelbrot | |
| --- | --- | --- | --- | --- |
| 3 | R =0.2936 | p =0 | R =0.2099 | p =0 |
| 4 | R =0.0407 | p =0 | R = -0.3630 | p =0 |
| 5 | R =0.3050 | p =0 | R = -0.1155 | p =0 |
| 6 | R =0.3846 | p =0 | R =0.0489 | p =0 |

**Supplementary Table 4:** Spearman correlations controlling for GC Content and model goodness of fit for the Truncated Power Law across k-mer lengths. In order to best demonstrate the shift in behavior, we calculate the Spearman correlation for the 4 quartiles of our samples. R denotes the Spearman correlation and all p values were found to be 0.

| k | Truncated Power Law | | | |
| --- | --- | --- | --- | --- |
|  | 1rst | 2nd | 3rd | 4th |
| 3 | R = -0.568 | R = -0.207 | R = -0.046 | R = 0.346 |
| 4 | R = -0.570 | R = 0.116 | R = -0.191, | R = 0.634 |
| 5 | R = -0.563 | R = -0.068 | R = -0.003 | R = 0.746 |
| 6 | R = -0.444 | R = 0.015 | R =-0.136 | R = 0.728 |

**Supplementary Table 5:** Spearman correlations controlling for GC Content and model goodness of fit for the Zipf-Mandelbrot across k-mer lengths. In order to best demonstrate the shift in behavior, we calculate the Spearman correlation for the 4 quartiles of our samples. R denotes the Spearman correlation and all p values were found to be 0.

| k | Zipf-Mandelbrot | | | |
| --- | --- | --- | --- | --- |
|  | 1rst | 2nd | 3rd | 4th |
| 3 | R = -0.612 | R = -0.102 | R = -0.068 | R = 0.308 |
| 4 | R = -0.201 | R = 0.333 | R = -0.265 | R = 0.197 |
| 5 | R = -0.629 | R = 0.301 | R = -0.205 | R = 0.553 |
| 6 | R = -0.674 | R = 0.190 | R = -0.167 | R = 0.622 |

**References**

1. [Chen, F. *et al.* Complete Genome Sequence of Porcine Circovirus 2d Strain GDYX. *Journal of Virology* **86**, 12457 (2012).](http://paperpile.com/b/EoaZeV/5qEW)

2. [Fernández, P. *et al.* A 160 Gbp fork fern genome shatters size record for eukaryotes. *iScience* **27**, 109889 (2024).](http://paperpile.com/b/EoaZeV/7cgb)

3. [Moeckel, C. *et al.* A survey of k-mer methods and applications in bioinformatics. *Comput Struct Biotechnol J* **23**, 2289–2303 (2024).](http://paperpile.com/b/EoaZeV/gc1I)

4. [Mouratidis, I. *et al.* kmerDB: A database encompassing the set of genomic and proteomic sequence information for each species. *Comput Struct Biotechnol J* **23**, 1919–1928 (2024).](http://paperpile.com/b/EoaZeV/WdEW)

5. [Yang, Z. *et al.* Intrinsic laws of k-mer spectra of genome sequences and evolution mechanism of genomes. *BMC Evolutionary Biology* **20**, 1–15 (2020).](http://paperpile.com/b/EoaZeV/TqGtQ)

6. [Chor, B., Horn, D., Goldman, N., Levy, Y. & Massingham, T. Genomic DNA k-mer spectra: models and modalities. *Genome Biol* **10**, R108 (2009).](http://paperpile.com/b/EoaZeV/zYLV5)

7. [Ferrer-I-Cancho, R. & Forns, N. The self-organization of genomes. *Complexity* **15**, 34–36 (2010).](http://paperpile.com/b/EoaZeV/0WuZ)

8. [Linguistic laws in biology. *Trends in Ecology & Evolution* **37**, 53–66 (2022).](http://paperpile.com/b/EoaZeV/IrUo)

9. [Luscombe, N. M., Qian, J., Zhang, Z., Johnson, T. & Gerstein, M. The dominance of the population by a selected few: power-law behaviour applies to a wide variety of genomic properties. *Genome Biol* **3**, RESEARCH0040 (2002).](http://paperpile.com/b/EoaZeV/j2Ln)

10. [Furusawa, C. & Kaneko, K. Zipf’s law in gene expression. *Phys Rev Lett* **90**, 088102 (2003).](http://paperpile.com/b/EoaZeV/6k44)

11. [Mantegna, R. N. *et al.* Linguistic features of noncoding DNA sequences. *Phys. Rev. Lett.* **73**, 3169–3172 (1994).](http://paperpile.com/b/EoaZeV/OunK)

12. [Li, W. Zipf’s Law everywhere. *Glottometrics* (2002).](http://paperpile.com/b/EoaZeV/RPre)

13. [Konopka, A. K. & Martindale, C. Noncoding DNA, Zipf’s law, and language. *Science* **268**, 789 (1995).](http://paperpile.com/b/EoaZeV/W55E)

14. [Chatzidimitriou-Dreismann, C. A., Streffer, R. M. F. & Larhammar, D. Lack of Biological Significance in the ‘Linguistic Features’ of Noncoding DNA—A Quantitative Analysis. *Nucleic Acids Res* **24**, 1676–1681 (1996).](http://paperpile.com/b/EoaZeV/h9Uj)

15. [Sheinman, M., Ramisch, A., Massip, F. & Arndt, P. F. Evolutionary dynamics of selfish DNA explains the abundance distribution of genomic subsequences. *Scientific Reports* **6**, 1–8 (2016).](http://paperpile.com/b/EoaZeV/U4zU)

16. [Greg Warr, L. H. The Architecture of the Genome Integrates Scale Independence with Inverse Symmetry. *Academia Molecular Biology and Genomics* (2025) doi:](http://paperpile.com/b/EoaZeV/5utK)[10.20935/AcadMolBioGen7650](http://dx.doi.org/10.20935/AcadMolBioGen7650)[.](http://paperpile.com/b/EoaZeV/5utK)

17. [Li, W. Menzerath’s law at the gene-exon level in the human genome. *Complexity* **17**, 49–53 (2012).](http://paperpile.com/b/EoaZeV/3gIM)

18. [Chacoma, A. & Zanette, D. H. Heaps’ Law and Heaps functions in tagged texts: evidences of their linguistic relevance. *R Soc Open Sci* **7**, 200008 (2020).](http://paperpile.com/b/EoaZeV/8Ill)

19. [Tettelin, H., Riley, D., Cattuto, C. & Medini, D. Comparative genomics: the bacterial pan-genome. *Curr Opin Microbiol* **11**, 472–477 (2008).](http://paperpile.com/b/EoaZeV/5BAK)

20. [Bonnie, J. K., Ahmed, O. Y. & Langmead, B. DandD: Efficient measurement of sequence growth and similarity. *iScience* **27**, 109054 (2024).](http://paperpile.com/b/EoaZeV/7oxX)

21. [Range-limited Heaps’ law for functional DNA words in the human genome. *Journal of Theoretical Biology* **592**, 111878 (2024).](http://paperpile.com/b/EoaZeV/Lk7w)

22. [Baeza-Yates, R., Glaz, J., Gzyl, H., Hüsler, J. & Palacios, J. L. *Recent Advances in Applied Probability*. (Springer Science & Business Media, 2006).](http://paperpile.com/b/EoaZeV/gkkU)

23. [Nguyen, E. *et al.* Sequence modeling and design from molecular to genome scale with Evo. *Science* (2024) doi:](http://paperpile.com/b/EoaZeV/Ltdb)[10.1126/science.ado9336](http://dx.doi.org/10.1126/science.ado9336)[.](http://paperpile.com/b/EoaZeV/Ltdb)

24. [Koulouras, G. & Frith, M. C. Significant non-existence of sequences in genomes and proteomes. *Nucleic Acids Res* **49**, 3139–3155 (2021).](http://paperpile.com/b/EoaZeV/rpOM)

25. [Georgakopoulos-Soares, I., Yizhar-Barnea, O., Mouratidis, I., Hemberg, M. & Ahituv, N. Absent from DNA and protein: genomic characterization of nullomers and nullpeptides across functional categories and evolution. *Genome Biology* **22**, 1–24 (2021).](http://paperpile.com/b/EoaZeV/Fko7)
